# Spectral-Phase Interferometry Detection of Ochratoxin A via Aptamer-Functionalized Graphene Coated Glass

**DOI:** 10.3390/nano11010226

**Published:** 2021-01-16

**Authors:** Nikita Nekrasov, Natalya Yakunina, Averyan V. Pushkarev, Alexey V. Orlov, Ivana Gadjanski, Amaia Pesquera, Alba Centeno, Amaia Zurutuza, Petr I. Nikitin, Ivan Bobrinetskiy

**Affiliations:** 1 National Research University of Electronic Technology, 124498 Moscow, Russia; 8141147@gmail.com (N.N.); natali.swan.1999@mail.ru (N.Y.); 2 Moscow Institute of Physics and Technology, 9 Institutskii per., Dolgoprudny, 141700 Moscow, Russia; pushkarev@phystech.edu (A.V.P.); alexey.orlow@gmail.com (A.V.O.); 3 Prokhorov General Physics Institute of the Russian Academy of Sciences, 38 Vavilov St, 119991 Moscow, Russia; petr.nikitin@nsc.gpi.ru; 4 BioSense Institute-Research and Development Institute for Information Technologies in Biosystems, University of Novi Sad, 21000 Novi Sad, Serbia; igadjanski@biosense.rs; 5 Graphenea, Avenida de Tolosa 76, 20018 Donostia-San Sebastián, Spain; a.pesquera@graphenea.com (A.P.); a.centeno@graphenea.com (A.C.); a.zurutuza@graphenea.com (A.Z.)

**Keywords:** CVD graphene, label-free biosensing, mycotoxins, aptamer, spectral-phase interferometry

## Abstract

In this work, we report a novel method of label-free detection of small molecules based on direct observation of interferometric signal change in graphene-modified glasses. The interferometric sensor chips are fabricated via a conventional wet transfer method of CVD-grown graphene onto the glass coverslips, lowering the device cost and allowing for upscaling the sensor fabrication. For the first time, we report the use of graphene functionalized by the aptamer as the bioreceptor, in conjunction with Spectral-Phase Interferometry (SPI) for detection of ochratoxin A (OTA). In a direct assay with an OTA-specific aptamer, we demonstrated a quick and significant change of the optical signal in response to the maximum tolerable level of OTA concentration. The sensor regeneration is possible in urea solution. The developed platform enables a direct method of kinetic analysis of small molecules using a low-cost optical chip with a graphene-aptamer sensing layer.

## 1. Introduction

Development of label-free, highly sensitive, direct, and real-time-enabled optical biosensors for small molecules (less than 1 kDa in size) is a challenging task of high importance in many areas of health analysis, environmental and food control, agriculture, etc. [1]. In order to develop and fabricate such biosensing devices, it is necessary to combine outstanding methodology, instrumentation, specific recognition elements, and immobilization strategies. Nanomaterials are becoming one of the most promising tools for sensing of the small molecules due to their strong response based on the physical or chemical principles. Graphene, in particular, as a single-layer atomic surface, suggests a highly sensitive technology for single molecule detection based on electrical or optical methods, which can be performed in a low-cost manner with high potential scalability [2].

In the last decade, the graphene-coated surface plasmon resonance (SPR) interfaces have been actively studied for application in biosensing [3,4]. Graphene, having high affinity to biomolecules, provides high adhesion and signal amplification. In addition, graphene’s optical property alters the surface plasmon properties, and, thereby, increasing sensitivity to the refractive index change. This phenomenon is more pronounced for the biosensors based on multi-layered graphene [3,5,6]. Recently, major efforts have been applied in order to combine graphene with SPR [3,7]. Intrinsic plasmons in graphene, which are tunable by the environmental changes, offer a variety of potential applications [8]. An important application of graphene-based and graphene-SPR combined sensors is the food quality assessment, particularly concerning the presence of mycotoxins in foodstuffs [9,10,11].

Ochratoxin A (OTA) is one of the most frequently occurring mycotoxins found in a number of different food products, including meat, meat products, and edible offal [12]. In the context of the project REALSENSE1 (www.realsense.rs), aiming to develop sensors for optimization of a cultivated meat bioprocess, it is important to consider sensing options for mycotoxins as well, including OTA.

The cultivated meat bioprocess starts with the seed train of primary cells, obtained by a biopsy from the animal. Since the animal may have been already fed with mycotoxin-contaminated feed [13], it is important to have tools to make sure the harvested cells are mycotoxin-free.

Detecting the changes in optical properties such as absorption, reflection, and an interferometric pattern in the presence of analytes is one of the prospective methods for mycotoxin detection. Recently, graphene-based refractive index optical sensors were demonstrated for detection of different analytes [14,15]. The relatively high intra-band optical absorption in graphene can greatly degrade the optical resonance in the sensors based on micro-ring resonators and photonic crystals, demanding for optical configuration adjustment and, thus, greatly increasing the complexity and the price of the technology [16].

However, interferometric methods are less sensitive to graphene integration and can provide easy functionalization of the sensing area [17], yielding a low-cost and relatively simple biosensing device. Interferometric biosensing is based on a comparison of the optical changes in interference of the control and the sensing beams produced by the biorecognition event in the sensing area or on phase changes between s- and *p*- polarizations of a single beam [18]. Bioreceptors immobilized on the sensor surface help to increase the sensor’s selectivity and sensitivity. Various bioreceptors can be utilized, such as antibodies, enzymes, and aptamers, with the latter recently emerging as one of the most promising, for several reasons. Aptamers are short synthetic DNA or RNA molecules whose structural conformation allows them to bind target molecules with high specificity and sensitivity [19]. Aptamers are readily synthesized by the SELEX method and, owing to their small size, high chemical stability, and thermal stability, as well as low price, aptamers can be a good alternative for antibodies [1]. However, a disadvantage of aptamers in the context of detection of mycotoxins and other small molecules is their size and associated small thickness change upon analyte binding (less than 1 nm), which is why the direct assay optical methods can result in a low sensitivity compared to antibody-based methods or methods based on competitive assays [20,21]. It is quite challenging to perform direct assay of mycotoxin detection based on polarization interferometry using aptamers [22,23].

Most commonly, interferometric sensors use the competitive immunoassay format or the binding inhibition assay where the target is attached to the surface [1,24]. Various interferometric methods, such as polarization interferometry, were applied for small molecules’ detection based on aptamer recognition elements [25]. The highly sensitive recognition with the detection limit down to 0.7 pg/mL was demonstrated. A disadvantage for upscaling is the fact that the chip preparation in the described study was still based on complicated micro-technological and nanotechnological process for planar optical waveguides production.

In this work, we report, for the first time, the label-free optical detection of ochratoxin A (OTA) in a direct assay using graphene functionalized with the OTA-specific aptamer and implementing the method of spectral phase interference (SPI). The SPI method is based on measuring the phase shift in an interference pattern produced by the reflected light from different surfaces within the sensor chip [26]. This method was demonstrated to be highly effective for detection of the small molecules in competitive assays. In the current work, we aim to investigate how the optical properties of the sensor chip change when small molecules absorb on its surface in direct conjugation with aptamers. Graphene is placed on the sensing side of the glass slip to ensure the covalent bonding of the aptamer to the surface and to increase the sensitivity to OTA bioconjugation. In particular, we set to investigate the effect of the change of the optical properties of the sensor chip after OTA binds to the aptamer on its surface. The results demonstrate prominent and highly measurable optical signals of the graphene-coated glass sensor in response to the tolerable level of OTA concentration of 4 ng/mL with the assay time of 6 min using a compact biosensor powered by a notebook USB port.

## 2. Materials and Methods

### 2.1. Graphene Optical Chip Fabrication

Single-layer graphene chemical vapor deposited (CVD) on 25-µm thick copper foil was provided by Graphenea (Spain). A poly (methyl methacrylate) (950PMMA A4, Ithaca, NY, USA) was spin-coated with a step-wise increase of the speed from 1500 to 2500 rpm for 1 min on top of the graphene/copper stack and used as a support layer during the transfer. The stack was baked on a hot plate at 150 °C for 5 min. We cut the copper/graphene/PMMA stack to pieces of about 4 × 5 mm^2^ size. The graphene from the backside of the copper foil was etched in solution made of deionized (DI) water, hydrochloric acid (30%), and hydrogen peroxide solution (1:2:20, HCl/H_2_O_2_/ H_2_O). To remove the copper foil, the sample was etched in aqueous solution of iron (III) chloride for about two hours. Prior to graphene transfer, the surface of the glass cover slips was washed in boiled dimethylformamide (DMF) and activated in oxygen plasma for 5 min. PMMA/graphene stack was rinsed in deionized (DI) water, and transferred onto the glass cover slip. A chip was left under ambient conditions overnight to dry out. To reflow the PMMA and improve the graphene-to-substrate adhesion, we annealed the chip at 150 °C for 5 min [27]. Afterward, the PMMA was dissolved in DMF (three times for 15 min in 150 °C). Finally, the structure was washed with DI water, dried under nitrogen flow, and annealed at 200 °C for 30 min to remove residual water. Prior to the sensor assembly, the surface of the graphene chip was UV-treated (low-pressure mercury lamp Svetolit-50 (LIT, Moscow, Russia)) at normal conditions for 4 min to remove any organic residuals [28].

### 2.2. Aptamer Immobilization on the Sensor Chip

Aptamer immobilization on graphene was performed in a way previously described for graphene-based transistor [10]. 100 µL drop of 1 mM solution of 1-Pyrenebutyric acid *n*-hydroxysuccinimide ester (PBASE) in dimethylformamide (DMF) was spread over the cover glass for 6 h in order to activate the graphene surface. Afterward, the glass was washed thoroughly with DMF, isopropanol (IPA), and DI water for 3 min each. At the last step, the glass was gently blown with the air gun. The OTA aptamer with sequence of GAT CGG GTG TGG GTG GCG TAA AGG GAG CAT CGG ACA [29,30] with amino modified 5′ end and purified by HPLC was purchased from Metabion AG (Planegg, Germany) and dissolved in PBS buffer (pH 7.4). Covalent bonding of OTA aptamer to the PBASE *via* an *n*-hydroxysuccinimide cross-linking reaction [31] was carried out in the 3 µM solution for 12 h in a humid atmosphere to prevent the solution evaporation. This was followed by washing with PBS solution baths (2 min each) and drying with the air gun for ~1 min. A 100 mM ethanolamine (ETA) in PBS was applied for 1 h to deactivate and block the excess reactive groups on the graphene surface. Excessive ETA was removed by the washing in PBS and DI water.

### 2.3. Graphene Chip Characterization

Raman spectra were recorded on the Centaur HR microRaman spectrometer (Nanoscan Technology, Dolgoprudnyy, Russia) with a 100× objective at a 532-nm wavelength (Cobolt, Solna, Sweden) with a beam spot of ≈1 μm^2^ and laser power of 0.5 mW. A Solver Pro atomic force microscope (AFM) (NT-MDT, Moscow, Russia) was used to study the morphology of the graphene transferred onto the cover slip. The silicon semi-contact cantilevers NSG03 (Tiposnano, Estonia) with resonance frequency of 105 kHz were used in order to avoid any damage to the graphene during characterization.

### 2.4. Measurements in Mycotoxin Solution

To investigate the effect of the small molecules’ conjugations with the modified graphene, we dissolved the OTA solution (10 µg/mL in acetonitrile, Sigma-Aldrich, St. Louis, MO, USA) in PBS in different concentrations. We used a compact single-channel label-free biosensor without thermal stabilization based on the spectral-phase interferometry, which was powered and controlled via a notebook USB port [32] (GPI RAS, Moscow, Russia). Briefly, the method employs a super-luminescent 850-nm diode radiation incident on a microscope cover slip that serves both as a biochip and as a two-beam interferometer. Interference between a beam reflected from the bottom surface of the sensor chip and a beam reflected from the upper surface of the sensor is observed. The changes Δd in the optical thickness of the slip with the sensing layer are determined in the analyzed spot on the sensor chip by measuring the phase shift of the interference pattern. Upon adsorption/desorption of the biomolecules and during conformational changes of the sensing layer, the optical thickness of the slip with the graphene layer changes. The value Δd is averaged over the registration spot area with a diameter of 1 mm. The temporal dependence of Δd (sensogram) is recorded in real time throughout an experiment. The solution is pumped to the sensing layer by the peristaltic pump with controlled speed.

## 3. Results and Discussion

**Figure 1:**
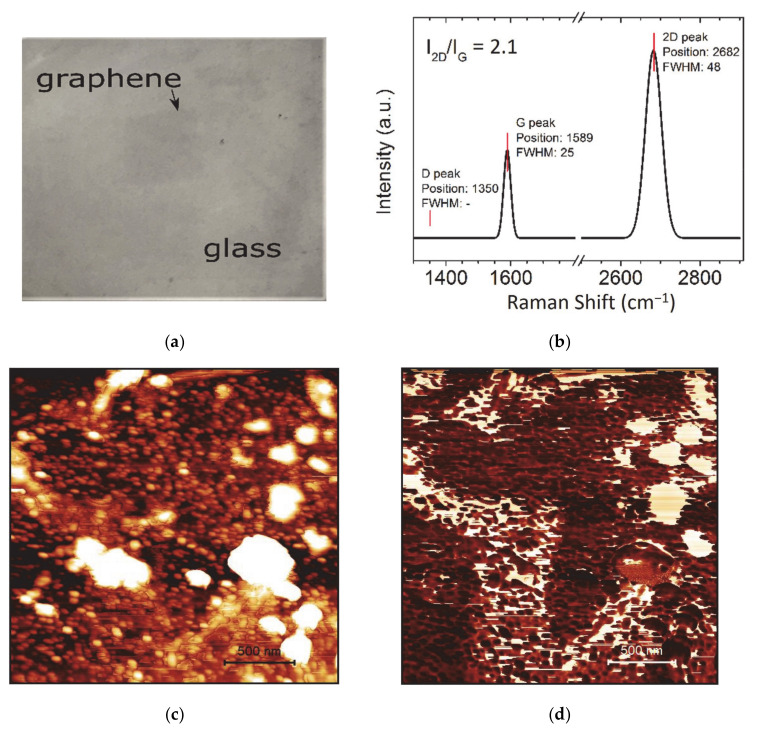
The sensor chip based on the glass/graphene stack. (**a**) Photography of the sensor chip. Image contrast enhancement was applied. (**b**) Raman spectra of graphene on glass. Atomic force microscopy image of graphene on glass: (**c**) height image and (**d**) phase shift contrast. Scale bar: 500 nm.

The graphene/glass stack was characterized by atomic force microscopy and Raman spectroscopy. Graphene has a very low absorption for the visible light of only 2.3%, which makes the graphene monolayer on the glass cover slip very transparent and difficult for visual inspection without proper interference (see Figure 1a). Hence, the quality of the graphene transferred onto the glass was controlled by the Raman spectroscopy using a ratio of G and D bands (Figure 1b). The 2.1 ratio for 2D to G intensity confirms the high quality of transferred graphene monolayer. This is further supported by the low intensity of the D band, responsible for defects in a hexagonal atomic lattice. The cover slip glass has relatively high roughness of about 5 nm, confirmed by the AFM (Figure 1c). The appearance of the nanometer-sized grains on the glass surface with dimensions up to 100 nm can increase the defects in graphene during transfer that might alter the reproducibility of the sensors’ properties in chip-to-chip fabrication. The phase shift of the cantilever oscillations can provide more informative data on the cracks in graphene, which are rather small (Figure 1d). These cracks should not affect the sensor’s response, since they are integrated by the signal collected in the area of the light spot with a diameter of 1 mm.

**Figure 2:**
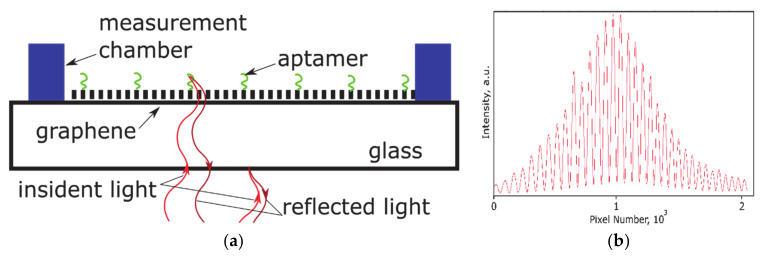
(**a**) Scheme of the interference pattern formation in glass cover slip/graphene with aptamer layer on the sensing side. (**b**) Reflection spectrum of a glass cover slip with graphene on the top side.

The scheme of interference of the light waves after reflection from the referent and the sensitive side of the glass is shown in Figure 2a. The SPI method is based on the secondary light waves produced after reflection on the referent and the sensitive side and the modulated intensity distribution in the spectrum of the reflected light that strongly depends on the phases of these waves [26]. The sensitivity of this method is less affected by the surface roughness in comparison to the recently proposed method based on total internal reflection measurements [33]. Taking into account that the reflective index for graphene (*n* = 2.9 at 850 nm [34]) is relatively high compared to the indices of air (1), glass (1.5), and water (1.33), its effect on the phase shift cannot be neglected. Figure 2b shows the reflection spectrum measured by the linear charge-coupled device (CCD) for cover glass/graphene stack illuminated by an IR light source with a central wavelength of 850 nm and a spectral width of 30 nm (at half maximum).

**Figure 3:**
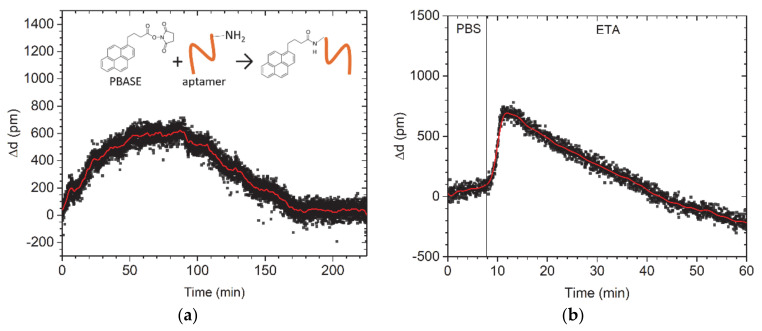
Assembling of bioreceptors on the graphene/glass stack. Sensogram showing the course of (**a**) aptamer immobilization on graphene by a chemical reaction with PBASE and (**b**) ETA deposition for deactivation of unreacted groups after aptamer deposition. Insert at (**a**): scheme of covalent bonding of an amine-modified aptamer to PBASE.

We employed the possibility of the measuring system to follow the kinetics of the binding process to control the aptamers’ immobilization on graphene functionalized by the π-π stacked PBASE. The glass/graphene stack was exposed overnight to a 300-nM OTA aptamer solution in PBS (Figure 3a). The immobilization process is very slow due to the covalent bonding of the amine group on the 5′ end of the aptamer, which requires proper orientation of the aptamer. If the aptamer is physically adsorbed on the graphene surface without covalent bonding, it will be removed at the washing step, leaving the PBASE group active. In order to decrease the nonspecific reaction after aptamer deposition, we applied the ETA to block the formation of the excessive reactive groups. The pumping process was set to a slow speed of 0.5 µL/min. However, the turbulence of the flow may also additionally decrease the efficiency of the bonding process. We observed unusual thickness modulation during aptamer deposition that can be related to the specific changes in aptamer interaction with the chip optical properties. In addition, during the immobilization, two simultaneous processes may take place, namely: (i) thickness increase as a result of aptamer binding, and (ii) thickness reduction caused by orientational and conformational relaxation of the immobilized aptamers. These two processes have different kinetic characteristics, which is the reason why the binding cannot be described by a single exponential dependence (Figure 3a). ETA molecules do not have a significant effect on the thickness of the layer. However, ETA, as a weak base, can affect the optical energy band of graphene via pH changing. The clear height shift in the sensogram (Figure 3b) can be explained by the significant change of the optical properties of the highly concentrated ETA solution. Partial washing out of the aptamer molecules, which did not bond to the graphene, may be responsible for decreased thickness during the blocking process.

In this research, we used the OTA mycotoxin (403.8 Da [35]) as an example of a small molecule. As a proof-of-concept of the method, we used the OTA concentration of 10 nM in PBS that corresponds to the general maximum tolerable level of toxin concentration in food [36].

**Figure 4:**
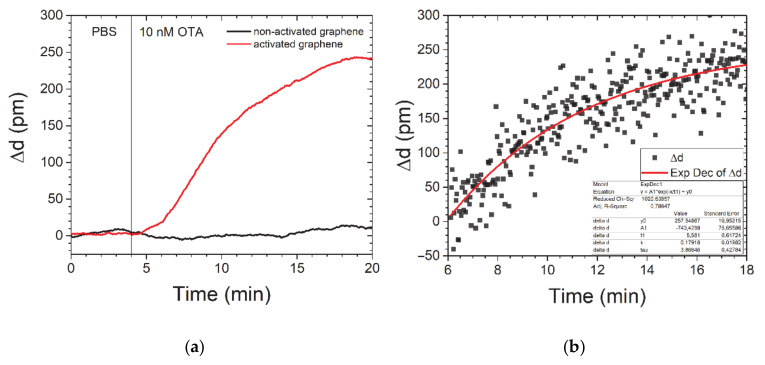
Sensogram of the course of 10-nm OTA detection for pristine and aptamer-modified graphene (**a**) and data fitting to an exponential function for a time constant calculation (**b**). In (**a**), we applied an adjacent-averaging method for pristine graphene curves to decrease the noise impact.

Figure 4a shows the results of the SPI data analysis for the thickness changes after adding the OTA. The increase of graphene thickness corresponds to the increase of aptamer layer thickness upon binding of OTA. This result is opposite of the negative thickness change previously observed for the SPR sensor with an active gold layer [35]. Such a difference is explained by the different adsorption behaviour of aptamers on gold and graphene layers. While graphene provides strong adsorption of DNA by π-π stacking of nucleotides [37], the gold or glass substrates have weak binding energy to DNA, leaving it free-standing. Importantly, OTA aptamers are guanin-rich and have been shown to fold in the anti-parallel G-quadruplex after binding to the OTA [38]. Thus, upon OTA binding to the aptamer, the folded G-quadruplex will increase the effective thickness on the graphene layer. It is possible that the OTA binding to the aptamer also induces the reflective index change [14], but the signal may be too low for the current configuration of the measurement set-up. Notably, we did not observe any response after OTA application to the pristine graphene on glass, i.e., not functionalized with the aptamer.

The response time *τ* was calculated from the time course of the sensogram for the 10-nM concentration of OTA (Figure 4b) based on the equation below [39].

(1) $$R\left(t\right)=R_{max}\left(1-e^{-\frac{t}{\tau }}\right),$$

where time constant *τ* is depending on toxin concentration and rates of association and dissociation of the aptamer and OTA. The results of binding kinetics fitting according to Formula (1) give an approximation of *τ* ≈ 6 min for 10 nM, which is about 40% lower than previously recorded data for the OTA aptamer by methods of total internal reflection ellipsometry (TIRE) [35]. In Table 1, we summarised the recent progress in OTA detection utilizing aptamers as bioreceptors. There is still a lack of methods for OTA detection that implement the cost-effective design of the sensing elements and can be performed in a multiplex format. The significant change of the optical signal in response to the maximum tolerable level of OTA concentration demonstrated in the current research is a promising method for rapid screening of OTA in the food samples and may be further developed to include the multiplexing approach.

Between measurements, the sensor was regenerated by washing in urea solution. We observed a nonlinear response to OTA concentration with the limit of detection of OTA above 1 nM. The thickness changes occurring upon OTA binding to the aptamer are presumed as the main mechanism of the sensor response. The shift in Fermi level under adsorption or reconfiguration of molecules (aptamer) can be interfered by the thickness change in absorbing molecules as the aptamer transforms from the linear stack to the G-quadruplex [29] with an estimated size up to several nm. We suggest that device sensitivity to the toxin in a direct assay is increased when nearly 100% of the aptamer sites are occupied, which corresponds to ~10 nM concentration [10].

However, the response of graphene to small molecules can be even more complex. When the aptamer molecule is attached to the graphene, it will increase electrostatic doping by the negatively charged aromatic bases of the nucleic acid in the aptamer molecule. Furthermore, the conformational change of the aptamer after bonding to the surface leads to decrease in electrostatic doping and, hence, change in the conductivity of graphene [10]. These changes can exert strong influence on the effective refractive index of the glass-graphene interface, resulting in the spectral changes of the interferometric phase signal [41]. As previously discussed, the refractive index of a small molecules layer is not significantly affected by the aptamer reconfiguration [35]. Nevertheless, using the thicker layer on graphene (e.g., rGO layers), one can increase the sensitivity to the refraction index changes, thus, decreasing the limit of detection [6,14]. In addition, demonstrated direct detection of mycotoxin by aptamers appears to be more convenient than a traditional competitive assay for the multiplex analysis of food products for the presence of several contaminants simultaneously. For this purpose, a modification of SPI for spectral-correlation interferometry for multiplex biosensing with reference channels [42] can be used.

## 4. Conclusions

In conclusion, we have developed a proof-of-concept for the robust and scalable methodology based on the interferometric optical sensors for direct, label-free detection of the small molecules, such as mycotoxins by aptamer on a glass surface. We prepared the cover glass/graphene stack as a sensor chip with the OTA-aptamer covalently bonded onto the graphene surface. Implementing a compact interferometric biosensor powered and controlled via a notebook USB port, we demonstrated the sensitivity on the level of 10 nM of OTA with an assay time of 6 min. This technology is very flexible and can provide the basis for a direct assay of various small molecules’ detection based on aptamer-functionalized graphene, potentially in a multiplex format. Given that the interaction of aptamers and small molecules can be registered via changes of the graphene optical properties, it opens the way for utilization of the assay in drug discovery, food, and medical analyses. The described graphene-based measurement system is a potential new tool for cultivated meat contamination assessment as well.

## Figures and Tables

**Table 1:** Aptamer-based biosensors for ochratoxin A detection.

Detection Method	Assay	Response Time, min	Limit of Detection, nM	Rev
FET	Aptamer/graphene FET	5	0.01	[10]
fluorescence	Aptamer/ZnPPIX probe	-	0.03	[29]
TIRE	Aptamer/gold/glass	10	0.03	[35]
fluorescence	Aptamer/SWCNH	-	15	[40]
SPI	Aptamer/graphene/glass	6	1	This work

FET–field effect transistor. ZnPPIX–Protoporphyrin IX Zinc (II). SWCNH–single-walled carbon nano horn.

## Data Availability

The data presented in this study are available on request from the corresponding author.
